# A phase II clinical trial of a dental health education program delivered by aboriginal health workers to prevent early childhood caries

**DOI:** 10.1186/1471-2458-12-681

**Published:** 2012-08-21

**Authors:** Fiona Blinkhorn, Ngiare Brown, Ruth Freeman, Gerry Humphris, Andrew Martin, Anthony Blinkhorn

**Affiliations:** 1 School of Health Sciences, University of Newcastle, PO Box 127, Brush Road, Ourimbah, New South Wales, 2258, Australia; 2 Graduate School of Medicine, University of Wollongong, New South Wales, Australia; 3 Dental Health Services Research Unit, University of Dundee, Dundee, 2522, UK; 4 School of Medicine, University of St Andrews, Fife, Scotland, KY16 9TF, UK; 5 NHMRC Clinical Trials Centre, Sydney Medical School, University of Sydney, Sydney, New South Wales, 2006, Australia; 6 Population Oral Health, Faculty of Dentistry, University of Sydney, Sydney, New South Wales, 2006, Australia

**Keywords:** Oral health, Aboriginal families, Health promotion

## Abstract

**Background:**

Early Childhood Caries (ECC) is a widespread problem in Australian Aboriginal communities causing severe pain and sepsis. In addition dental services are difficult to access for many Aboriginal children and trying to obtain care can be stressful for the parents. The control of dental caries has been identified as a key indictor in the reduction of Indigenous disadvantage. Thus, there is a need for new approaches to prevent ECC, which reflect the cultural norms of Aboriginal communities.

**Methods/Design:**

This is a Phase II single arm trial designed to gather information on the effectiveness of a dental health education program for Aboriginal children aged 6 months, followed over 2 years. The program will deliver advice from Aboriginal Health Workers on tooth brushing, diet and the use of fluoride toothpaste to Aboriginal families. Six waves of data collection will be conducted to enable estimates of change in parental knowledge and their views on the acceptability of the program. The Aboriginal Health Workers will also be interviewed to record their views on the acceptability and program feasibility. Clinical data on the child participants will be recorded when they are 30 months old and compared with a reference population of similar children when the study began. Latent variable modeling will be used to interpret the intervention effects on disease outcome.

**Discussion:**

The research project will identify barriers to the implementation of a family centered Aboriginal oral health strategy, as well as the development of evidence to assist in the planning of a Phase III cluster randomized study.

**Trial registration:**

ACTRN12612000712808

## Background

Dental decay (caries) is a widespread disease within Aboriginal communities and it has a particularly severe impact on Aboriginal children. Recent health examinations conducted by medical and nursing personnel in the Northern Territory found early childhood caries to be the most common condition of Aboriginal children, affecting 43% of those examined [1]. In the context of the limited availability of dental personnel within many Aboriginal communities and due to the thin layer of enamel on primary teeth, dental caries can progress to the rapid destruction of the dentition. This is reflected in the high rates of hospitalisation of Aboriginal children requiring multiple dental extractions under paediatric general anaesthesia [2-4].

In recognition of the extent and severity of early childhood caries, its high hospitalisation rates and its impact on childhood nutrition, socialisation and schooling, the control of dental caries has been identified as a key indicator in the reduction of Indigenous disadvantage [5]. Yet the oral health of Aboriginal children is worsening rather than improving under current approaches, with increasing numbers of decayed, missing and filled teeth being found in this population. Indigenous children have twice the levels of dental caries in both their primary and permanent teeth compared with their non-Indigenous counterparts, as well as higher levels of untreated decay [6].

Many Aboriginal communities face this burden of oral disease with limited or no access to dental services, and current workforce projections predict a worsening shortage of dental personnel [7]. In addition few Aboriginal Controlled Community Health Services (ACCHS) are funded to provide dental care, and those that do have difficulty in attracting and retaining dental professionals, particularly in rural and remote Australia. Therefore there is an urgent need for the development of effective new approaches to prevent early childhood caries, which are appropriate to the needs of Aboriginal families and can be delivered within the limited resources of the ACCHSs.

After extensive consultation with a number of Aboriginal Controlled Community Health Service staff and the Director of the National Aboriginal Health College a promising family focused dental health education program has been developed. It will be delivered by Aboriginal Health Workers (AHSs) inviting Mothers with young children to an appropriate local ACCHS clinic to offer diet advice, ensure fluoride toothpaste is used on a regular basis and to screen children for early signs of early childhood caries. A home visit will be undertaken if families find it difficult to attend, miss an appointment or the AHW thinks a home visit is the best way to maintain contact with the family.

This is an innovative approach which responds to the severely limited availability of dental personnel within Aboriginal communities, the very early onset of the disease in Aboriginal children, and recognizes the importance of AHWs to local communities. Early childhood caries develops at a time when contact with dental personnel is very limited in comparison to the regular contact young families have with Aboriginal Health Workers. In addition the active recruitment to the program by trusted local AHWs will overcome the problem that individuals from deprived backgrounds are less likely to consent to targeted preventive intervention schemes [8,9].

The training program for AHSWs to explain how to implement the program has been tested and the supporting information leaflets have been piloted in collaboration with Aboriginal families. Both evaluations were positive in terms of their appropriateness and potential sustainability. The pilot data have been used to develop the protocol for a Phase II evaluation which will determine whether there is sufficient promise to warrant the investment in a large scale confirmatory Phase III cluster randomized trial. The development and evaluation of a program to improve oral health deliverable by community health personnel is relevant not only to Aboriginal communities, but also to other groups with limited access to dental services, including rural and remote communities throughout Australia.

## Methods/Design

The aim of the program of research is to address the need to improve the oral health of Aboriginal children [10-12]. The objective of the study is to build upon the success of the pilot work, using a phase II single arm trial design to gather preliminary evidence on the effectiveness of the program offered to Aboriginal children from six months of age who are at risk of dental caries. The program will be assessed over a period of 2 years to investigate not only the effectiveness, but also its feasibility and to inform the design of a subsequent confirmatory randomised phase III trial. The research follows the MRC framework for the design and evaluation of complex interventions to improve health [13]. The primary endpoint is the prevalence of early childhood caries at 30 months of age. Secondary endpoints include: changes in primary caregivers dental knowledge over time; number of episodes of dental care; impact of oral health problems on the family and qualitative measures of the program to assess its acceptability to the families. In addition the managers and Aboriginal Health Workers will be interviewed to record their views on the overall feasibility and sustainability of the program [14].

The pilot work and consultation with ACCHSs led to the development of the dental health education program which is characterized by the following strategies:

Delivery by trained Aboriginal Health workers;

Support for health oral behaviours offered to families with young children;

Focus on control of the night time bottle, toothbrushing and use of fluoride toothpaste;

Free toothpaste, and toothbrushes for all the family together with a ‘sippy’ (trainer) cup for the child;

Restricting the frequency of the consumption of sugary snacks and drinks.

Careful attention to the cultural appropriateness of teaching materials.

The development of the ACCHSs capacity to provide oral health support to vulnerable families through the development of the role of AHWs is based on Goodman’s model of organizational change [15].

The development of the oral health role of Aboriginal Health Workers is strongly supported by the National Aboriginal Community Controlled Health Organisation [16], the Australian Dental Association [17], Australia‘s National Oral Health Plan [18], and the National Aboriginal and Torres Strait Islander Oral Health Workshop [19]. Despite the extent of this support and the National Aboriginal Community Controlled Health Organization calling for the development of this role for over a decade there has been little sustainable development of the role of AHWs, and there has been no evaluation of the impact of such a role on the oral health of Aboriginal families. The disconnect between policy and practice in this area and the identification of facilitators and barriers to the development of this role has been the subject of recent research [20].

The program focuses on family/carers because they significantly influence whether a child will develop Early Childhood Caries (ECC) and will be responsible for establishing long lasting family norms of behaviour regarding tooth brushing behaviour and diet [21,22]. In order to support family/carers, AHWs will, systematically at predetermined intervals, provide them with oral health products, education materials, advice specific to the dental needs of the child and screen for early signs of ECC. This is an innovative approach to the prevention of oral disease in Aboriginal communities which contrasts the current model, whereby health education is delivered at irregular intervals.

The development of family knowledge and norms about the value and care of the primary dentition is important because many parents have the ‘commonsensical belief’ that if teeth do not hurt there are no dental problems [23,24]. Given this belief and the rapid progression of the disease, dental care may not be sought until the child requires hospitalization for dental extractions under general anaesthesia. Hence, the inclusion of oral health screening is an integral part of the program.

The value of strategies which focus on families with young children is further emphasised by research which has shown that a child with one carious lesion is five times more likely to develop a further carious lesion than a child with no dental caries [25]. This difference in risk is of major significance and demonstrates that early intervention to maintain a child’s oral health is the most logical and practical preventive strategy.

A lack of family carer access to dental health education and oral care products is a major barrier to maintaining a healthy mouth. Kruger et al [26] reports a significant lack of oral health awareness among Aboriginal people. The National Rural Health Alliance reports that 30 percent of Indigenous children do not own a toothbrush [27]. A study of Northern Territory Aboriginal communities reported that only 20 percent of children used toothpaste daily and most did not begin using toothpaste until the age of four [28]. The AIHW reports that ‘Less than 5 percent of remote Aboriginal and Torres Strait Islander pre-school children brush their teeth on a regular basis [6]. Causes identified for low use of toothbrushes and fluoride toothpastes include poor levels of carer education, high purchase costs and difficulties in establishing tooth brushing routines. The proposed program addresses these crucial barriers to the prevention of early childhood caries in Aboriginal families. AHWs will provide vulnerable families with advice on effective tooth brushing, use of fluoride toothpastes and support the development of these behaviours by the distribution of oral hygiene products and materials including toothbrushes, fluoride toothpaste and trainer cups [29]. These preventive strategies are based on high quality evidence derived from large population based randomized controlled trials and systematic reviews [30] and show a benefit when used over time. Therefore the theoretical construct underpinning the program is the tailoring of the oral health message to the specific oral health and psychosocial needs of Aboriginal families and their children [31].

The dental health education program and the research instruments have been piloted with Aboriginal families, Aboriginal Health Workers and aligns with the Aboriginal Health and Medical Research Council policies on partnerships with Aboriginal Communities.

The primary endpoint for the study will be:

The prevalence of ECC at the age of 30 months. These data will be compared with similar children aged 30 months participating in the Aboriginal communities in the first year of the program implementation.

The secondary endpoints include:

Changes in the oral health knowledge of the primary care giver over time.

Early referral for dental care, if the oral health screening by the Aboriginal health workers notes a problem.

Impact of the child’s oral health on the family.

Acceptability of the program recorded by interviewing parents and AHWs at the end of the program.

Sustainability assessed by interviewing managers of the participating Aboriginal Community Care Health Services.

### Study design

The study is designed to monitor a longitudinal oral health education program for Aboriginal children aged 6 months followed for 2 years. In order to assess the effect on dental caries, a sample size of 150 participants will provide at least 80 percent power at the two sided 95 percent level of significance to distinguish between a prevalence of 2 versus 1.6 ECC ( ECC includes decayed ,missing and filled primary teeth ) at 30 months allowing for as much as a 33% attrition rate (chosen following discussions with AHWs on population movement). The reference rate for ECC is informed by data provided by the Centre for Oral Health Strategy, New South Wales (personal communication and work by Jamieson et al [6]). The forward projection is that the participating children will have a reduction in the mean ECC score of 20 percent, to 1.6. This is highly plausible and is based on previous trials. For example a recent study where fluoride toothpaste was posted to families living in low socio-economic areas of Northern England reported a 16 percent reduction in dental caries in young children [32]. No personal advice was offered. An Australian study reported a 33 percent reduction in dental caries [33,34], following a health education program which included the application of topical fluoride.

The rejection of the null hypothesis will be considered indicative of a clinically significant benefit, and would provide a strong case for proceeding to a Phase III evaluation.

### Recruitment

Aboriginal Health Workers know their communities well, and which families are most in need of support. AHWs from six Aboriginal Medical Services (AMS) will each recruit 12 families with a child six months of age. Up to three AHWs per Aboriginal Medical service will work on the project giving a potential sample of 216. The recruitment strategies have been successfully piloted and AHWs were able to recruit 6 families within a week, so the suggested figure of 12 families per AHW for the main study is certainly reasonable. The AHWs will explain the project to parents/carers and be responsible for obtaining a signed consent form from participating families.

### Ethical approval

Approval to undertake this project was obtained from a number of Agencies, namely:

1. Royal Prince Albert Hospital Ethics Committee, Sydney, NSW, Australia. Reference Number – X09 – 0052 & HREC 09 / RPAH/ 85.

2. University of Newcastle Research Ethics Committee, NSW, Australia. Reference Number – H- 2012 – 0070.

3. AH & MRC Ethics Committee, NSW, Australia. Reference Number – 696 / 09.

### Dental health education program

**Figure 1:**
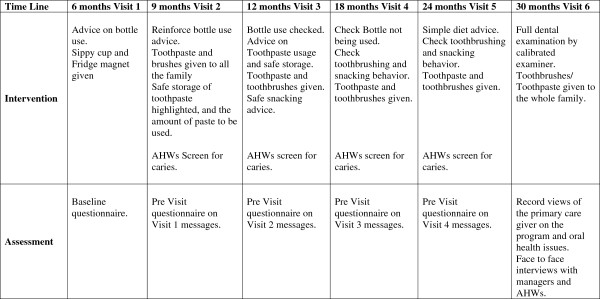
Dental health education program for Aboriginal families, beginning at six months and following on with advice from AHWs over time.

**Figure 2:**
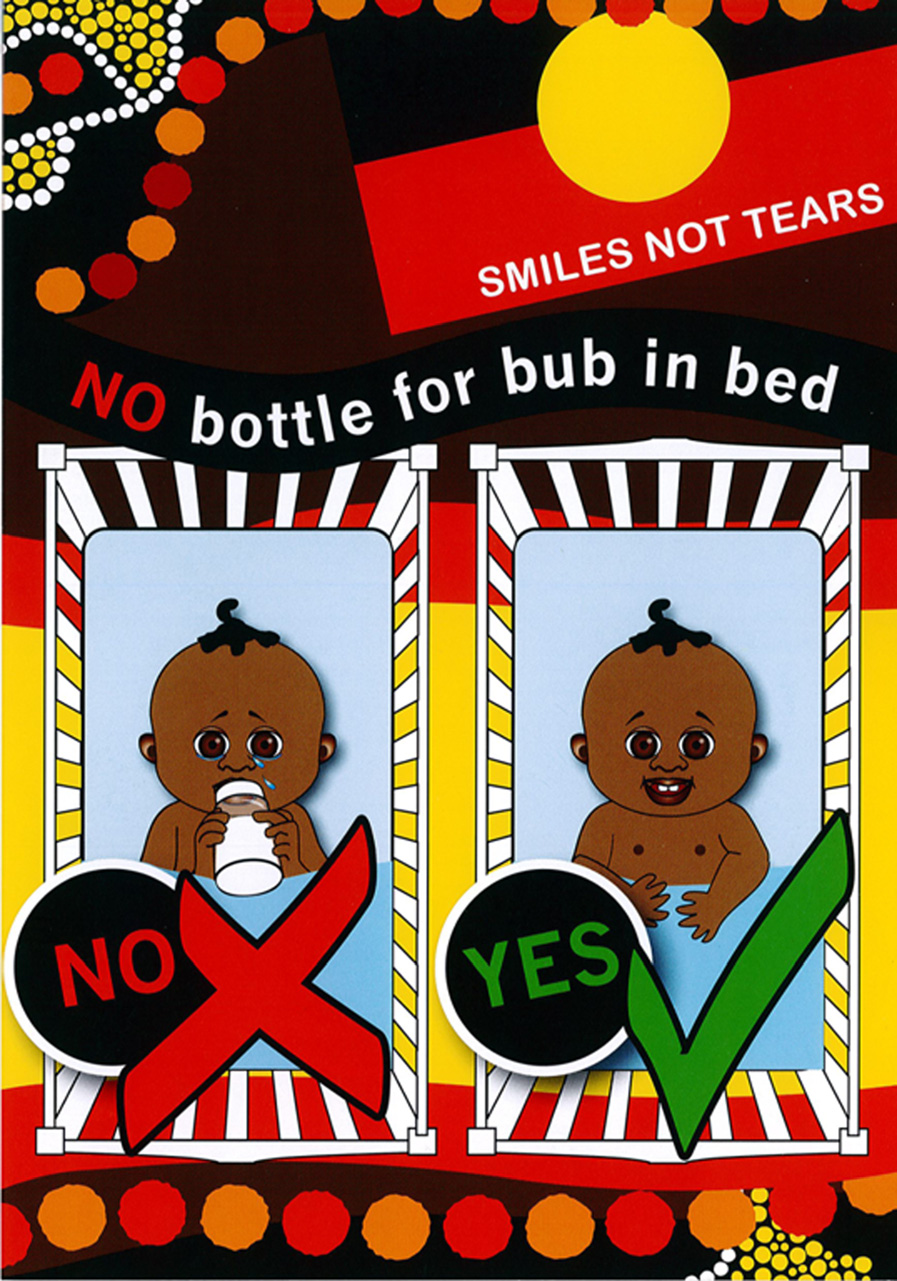
An Example of a leaflet to be given to Families participating in the program – focus on not using a night-time bottle.

The visit program is outlined in Figure 1 and centres on avoiding using a ‘bottle’ as a night –time comforter, using fluoride toothpaste from 9 months of age, encouraging the whole family to brush and having a simple oral health screening by the AHWs to assess whether early signs of dental caries are evident. Culturally appropriate leaflets and fridge magnets will be offered and an advice line is available should any of the families have specific problems. An example of one of the leaflets is shown in Figure 2.

### Data collection

Information on baseline and subsequent visits (Figure 1) will be collected by the AHWs utilizing a structured questionnaire which has been piloted in an Aboriginal community.

The key topics for intervention are:

Bottle use, when and what to put in it.

Tooth brushing – using the tooth brush

Toothpaste - using the toothpaste, how much to put on the brush and safe storage

Dietary habits – reported changes in the consumption of sugary snacks and drinks

AHWs screen for caries [35] – positive/negative

Reported dental problems

At the final 30-month assessment the carers will be asked about their perceptions of the feasibility, and acceptability of the program. In addition selected questions from the Dental Discomfort Scale [36] and the Being a Mother Scale [37] will be included in the assessment. The Aboriginal Health Workers and the senior management team at each AMS will be interviewed to record their views on the facilitators and barriers to the wider implementation of the program. All interviews will be recorded, transcribed and analyzed for recurrent themes.

The end of trial dental assessment will be undertaken by a calibrated examiner using the same diagnostic criteria when collecting the dental caries data on the historical reference group.

### Statistical analysis

The primary analysis will be an evaluation of the estimated prevalence of caries at 30 months in relation to the null hypothesis that the rate is 2. Descriptive analyses of other endpoints will be applied using methods appropriate for continuous and categorical data. Estimates will be presented with two-sided 95 percent confidence intervals.

The qualitative data from the interviews will be analyzed , using the ‘framework’ method [38]. The qualitative data will be fractured and examined for key topic areas. The data will then be systematically coded according to the topic areas and thematic charts (in spreadsheet form) will be constructed. Summaries of the relevant parts of transcripts will be written into the charts so that each case may be examined across a range of different and emerging themes [38].

The proposed study is rare in health promotion research in that it is not a ‘before and after’ design but is longitudinal, building information and capacity over time [39]. The longitudinal design helps the investigative team to test for mediation effects in a more rigorous manner than cross sectional designs [40]. For example the multi-wave nature of the assessments delivers the following methodological advances:

i. prevention on the reliance of retrospective reports of health behavior from mothers (removal of memory biases);

ii. reliable baseline latent variables of fluoride use and oral health (reducing bias of measurement error);

iii. enable the testing of growth models and unbiased parameter estimation of the fluoride intervention using resampling methods (bootstrapping). This third improvement would be made possible by controlling for concurrent baseline disease levels. Cole and Maxwell [39] suggest that Structural Equation Modelling (SEM) will greatly assist in testing the direct and indirect effects of a longitudinal intervention. Crucial is the development of a model which can reduce bias frequently inherent in two wave designs. These are sometimes referred to as ‘half-longitudinal’ [41]. The proposed design therefore has innovative features not utilized in previous caries prevention programs which will enable true longitudinal investigation benefiting from the latest developments in statistical modeling.

**Figure 3:**
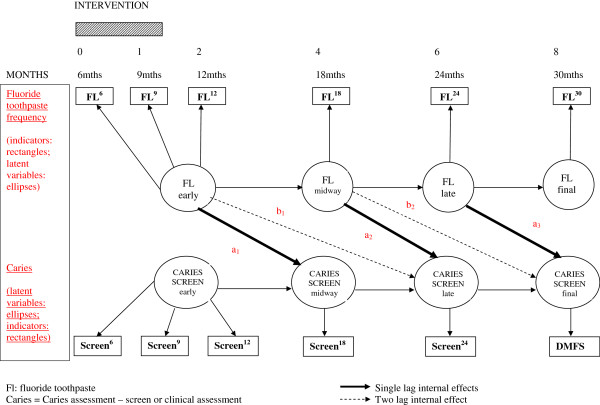
Potential Structural Equation Model.

One potential model which could be assessed utilizing Structural Equation Modeling is shown in Figure 3. The circles denote latent variable specification and the rectangles represent raw variables. Single indicator raw variables are permitted in SEM if the reliability estimate for the item is known. These estimates will be available from pilot work and previous published evidence. The arrows show theoretically derived effects specified for detailed examination in the statistical modeling. In addition, the model as depicted in Figure 3 can test for supplementary effects which are not possible in simple baseline and single post-intervention assessment designs.

Therefore the following will be tested:

i. the (lag 1) effect of fluoride intervention on the subsequent oral health assessment (parameters a_1_, a_2_ a_3_);

ii. a test of stability or equivalence of effect by constraining the parameters a_1_, a_2_ a_3_ (solid lines) to be equal and examining the significance of loss of ‘close fit [42]’.

iii. a test of the longer term effect (lag 2) of fluoride intervention beyond immediate subsequent oral health assessment controlling for lag 1 effects, as shown by parameters b_1_ and b_2_(dotted line).If these parameters are shown to be significant in this more complex model then an additional enquiry can be performed to test for the equivalence of these two lag 2 coefficients.

Although the health education intervention builds over 2 years the essentials of the appropriate health behaviours are built into the first 3 visits (shaded rectangle in Figure 3), controlling the night time bottle, reducing sugar consumption between meals, using a toothbrush, and applying fluoride toothpaste twice a day. Effectiveness is measured in terms of the caries screening and the final (30 month) full dental examination.

## Discussion

The proposed study has the potential to reduce dental caries in young Aboriginal children as the principal outcome focuses on a reduction in caries of 20 percent. This will have a major impact on the quality of life of Aboriginal families.

Aboriginal Community Controlled Health Services across Australia need evidence based strategies to improve the oral health of families under their care. These should be culturally appropriate, can be implemented by AHWs and deliver a proven benefit. Effective preventive programs are urgently required to reduce the burden of oral disease. The suggested dental health education program described in this paper has been carefully piloted and is culturally acceptable and does not require major resources to implement. Patterns of health behavior and lifestyle established in childhood are positive outcomes, building rather than trying to change daily oral health care habits. Parents and carers have a major influence on children’s behavior but are often not considered in early intervention activities. The program described may well contribute to Closing the Gap in terms of bringing some equity in oral health status between Aboriginal and Non-Aboriginal communities. The planned Phase II Trial includes multiple staged assessments of intervention inputs and defined primary and secondary outcomes. A carefully developed systematic model has been outlined with pre-determined statistically derived effects for testing the efficacy of the program. Such innovative programs require robust evaluation to influence and encourage all stakeholders and policy makers.

## Competing interests

The authors declare they have no competing interests.

## Authors’ contributions

FB prepared the health promotion program and under took pilot work. NB offered cultural advice and assisted with design of materials. RF advised on the health promotion program and assisted with the development of the protocol. GH gave statistical advice and helped with the theoretical framework. AM Statistical advice and developed clinical trial methodology. AB developed protocol and undertook pilot work. All authors read and approved the final manuscript.

## Pre-publication history

The pre-publication history for this paper can be accessed here:

http://www.biomedcentral.com/1471-2458/12/681/prepub
